# Supplementary material for: Chrysin Is Immunomodulatory and Anti-Inflammatory against Complete Freund’s Adjuvant-Induced Arthritis in a Pre-Clinical Rodent Model
Source: Pharmaceutics. 2023 Apr 12;15(4):1225. doi: 10.3390/pharmaceutics15041225 (PMC10144384; doi:10.3390/pharmaceutics15041225)
Supplement: Supplementary file 1 [file pharmaceutics-15-01225-s001.zip › pharmaceutics-2238164-supplementary.pdf]

Table S1. The sequence of oligonucleotides used to check mRNA expression levels.

Gene	Primer sequence (5′–3′)	Product size (bp)	Reference
TLR-2	Forward: CCA GAT GGC CAG AGG ACT CA	212	[36]
	Reverse: TGT GAG TCC CGA GGG AAT AGA		
NF-κB	Forward: CAT GGC AGA CGA CGA TCC TT	127	[65]
	Reverse: AAG GTA TGG GCC ATC TGT TGA		
TNF	Forward: ACA AGG CTG CCC CGA CTA T	67	[65]
	Reverse: CTC CTG GTA TGA AGT GGC AAA TC		
IL-4	Forward: AAC ACC ACG GAG AAC GAG CTC ATC	152	[66]
	Reverse: AGT GAG TTC AGA CCG CTG ACA CCT		
IL-10	Forward: AGA AGA GGG AGG AGC CTT TG	222	[66]
	Reverse: GCC TTT GCT GGT CTT CAC TC		
GAPDH	Forward: AGA GAC AGC CGC ATC TTC TT	494	[65]
	Reverse: TGC ATT GCT GAC AAT CTT GA		

References

36. Israr, M.; Naseem, N.; Akhtar, T.; Aftab, U.; Zafar, M.S.; Faheem, M.A.; Shahzad, M. Nimbolide attenuates complete Freund's adjuvant induced arthritis through expression regulation of toll-like receptors signaling pathway. *Phytother. Res.* **2023**, 37(3), 903-912.
65. Aslam, A.; Sheikh, N.; Shahzad, M.; Saeed, G.; Fatima, N.; Akhtar, T. Quercetin ameliorates thioacetamide-induced hepatic fibrosis and oxidative stress by antagonizing the Hedgehog signaling pathway. *J Cell Biochem.* **2022**, 123(8), 1356-1365.
66. Ahsan, H.; Irfan, H.M.; Alamgeer; Shahzad, M.; Asim, M.H.; Akram, M.; Zafar, M.S. (2021). Anti-rheumatic activity of pseudoephedrine (a substituted phenethylamine) in complete Freund's adjuvant-induced arthritic rats by down regulating IL-1β, IL-6 and TNF-*α* as well as upregulating IL-4 and IL-10. *Inflammopharmacology*. **2021**, 29(3), 673–682
